# Supplementary material for: Multi-Dimensional Elimination of β-Lactams in the Rural Wetland: Molecule Design and Screening for More Antibacterial and Degradable Substitutes
Source: Molecules. 2022 Dec 2;27(23):8434. doi: 10.3390/molecules27238434 (PMC9739631; doi:10.3390/molecules27238434)
Supplement: Supplementary file 1 [file molecules-27-08434-s001.zip › molecules-2048025-supplementary.pdf]

Supplementary File of Multi-Dimensional Elimination of β-Lactams in the Rural Wetland: Molecule Design and Screening for More Antibacterial and Degradable Substitutes

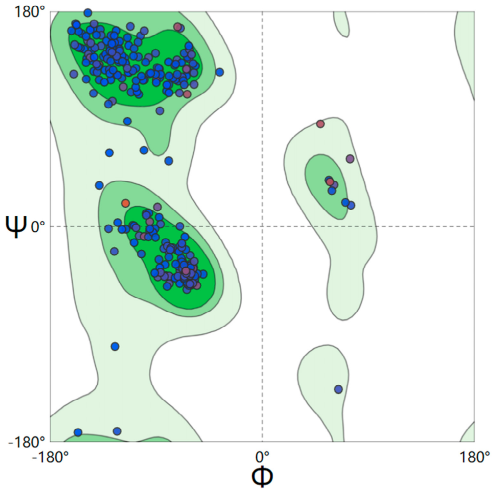

PBP-1

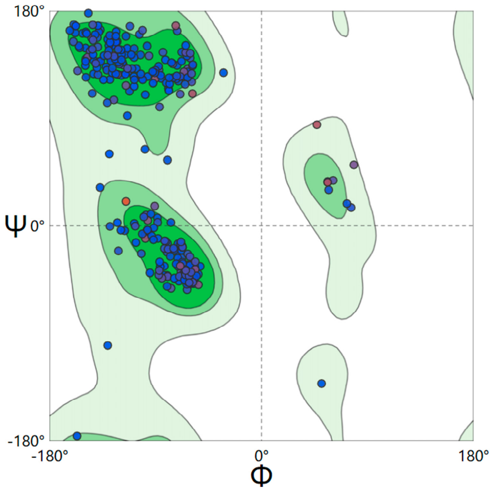

PBP-6

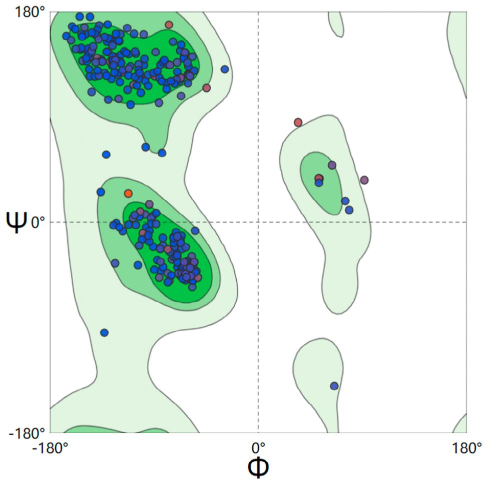

PBP-14

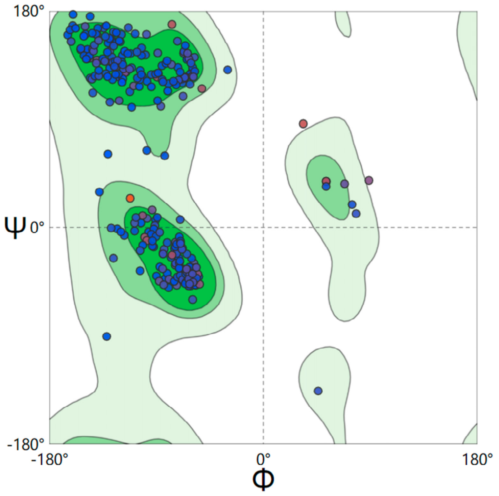

PBP-16

Figure S1 PBP resistant mutations Ramachandran map.

Table S1 LibDock Score and comprehensive evaluation values for degradability of β-lactams.

No.	Compounds	LibDock Score				
		6NTZ (Å)	4GZB (Å)	1Z98 (Å)	1B85 (Å)	CEI
**1**	(E)-Ceftriaxone	105.86	104.24	80.16	74.72	96.44
**2**	Aminoceftriaxone sodium	103.39	83.60	107.57	74.83	88.85
**3**	Amoxicillin	106.35	90.36	111.46	76.04	93.78
**4**	Ampicillin	103.97	91.70	101.15	73.03	92.16
**5**	Cefaclor	107.50	79.94	91.84	66.18	83.80
**6**	Cefadroxil	109.16	88.19	100.42	71.12	90.61
**7**	Cefalexin	107.76	89.12	93.69	68.20	89.46
**8**	Cefathiamidine	98.19	101.79	94.98	80.60	97.05
**9**	Cefazolin	97.07	106.88	106.64	91.07	103.00
**10**	Cefetamet	73.29	70.78	72.95	58.17	69.59
**11**	Cefixime	105.08	102.49	80.99	74.64	95.48
**12**	Cefmenoxime	102.34	100.10	89.03	85.37	96.57
**13**	Cefminox	102.24	98.71	105.65	79.01	97.33

**14**	Cefodizime	112.04	101.39	67.63	81.36	94.92
**15**	Cefotaxime	95.63	87.39	89.65	72.03	86.66
**16**	Cefotiam	114.70	106.21	82.10	87.35	101.04
**17**	Cefoxitin	87.60	85.27	104.10	72.60	86.54
**18**	Cefradine	82.27	80.14	95.19	63.44	80.21
**19**	Ceftizoxime	90.18	83.58	102.66	73.84	85.97
**20**	Ceftriaxone	96.43	103.92	93.96	80.82	97.84
**21**	Cefuroxime	98.43	87.85	87.39	66.43	86.16
**22**	Cephalothin (Cefalotin)	100.18	85.19	95.78	70.26	86.79
**23**	Loracarbef	101.23	80.81	97.20	71.10	84.88
**24**	Mecillinam	87.51	79.21	95.50	58.93	79.86
**25**	Oxacillin	97.10	69.95	80.77	56.15	73.58
**26**	Penicillin	70.00	81.70	95.11	67.49	79.83
**27**	PenicillinV	90.98	89.43	107.54	71.62	89.71

Table S2 Combined biodegradation prediction of β-lactam substitutes.

No.	Compounds	Pred.	Change rate (%)
-	PNC	79.83	-
PNC-1	PNC-18-N=NH_2	91.26	14.32%
PNC-2	PNC-18-N=NH_2-17-N_3	90.46	13.31%
PNC-3	PNC-18-N=NH_2-17-NH-NH_2	91.20	14.24%
PNC-4	PNC-18-N=NH_2-17-NO	91.33	14.40%
PNC-5	PNC-18-N=NH_2-17-NO_2	92.48	15.85%
PNC-6	PNC-18-N=NH_2-17-OCHO	90.80	13.74%
PNC-7	PNC-18-N=NH_2-17-ONO	91.08	14.09%
PNC-8	PNC-18-N=NH_2-17-OOH	90.53	13.40%
PNC-9	PNC-18-N=NH_2-17-SH	90.16	12.94%
PNC-10	PNC-18-N=NH_2-17-SO	91.52	14.64%
PNC-11	PNC-18-N=NH_2-17-SO_2	92.37	15.71%
PNC-12	PNC-18-N=NH_2-7-N=NH_2	91.08	14.10%
PNC-13	PNC-18-N=NH_2-7-N_3	90.80	13.74%
PNC-14	PNC-18-N=NH_2-7-NH-NH_2	91.41	14.51%

PNC-15	PNC-18-N=NH_2-7-NO	91.13	14.15%
PNC-16	PNC-18-N=NH_2-7-NO_2	91.98	15.21%
PNC-17	PNC-18-N=NH_2-7-OCHO	90.83	13.78%
PNC-18	PNC-18-N=NH_2-7-OOH	90.81	13.75%
PNC-19	PNC-18-N=NH_2-7-SH	89.76	12.44%
PNC-20	PNC-18-N=NH_2-7-SO	90.91	13.88%
PNC-21	PNC-18-N=NH_2-7-SO_2	90.98	13.96%
PNC-22	PNC-18-N_3	90.93	13.90%
PNC-23	PNC-18-NH-NH_2	92.25	15.55%
PNC-24	PNC-18-NH-NH_2-17-NO_2	87.66	9.81%
PNC-25	PNC-18-NH-NH_2-17-SO_2	87.59	9.71%
PNC-26	PNC-18-NO	91.45	14.55%
PNC-27	PNC-18-NO_2	96.13	20.42%
PNC-28	PNC-18-NO_2-17-N=NH_2	88.63	11.03%
PNC-29	PNC-18-NO_2-17-N=NH_2-7-N=NH_2	92.89	16.36%
PNC-30	PNC-18-NO_2-17-N=NH_2-7-N_3	92.85	16.31%
PNC-31	PNC-18-NO_2-17-N=NH_2-7-NH-NH_2	93.15	16.68%

PNC-32	PNC-18-NO_2-17-N=NH_2-7-NO	93.59	17.24%
PNC-33	PNC-18-NO_2-17-N=NH_2-7-NO_2	95.55	19.69%
PNC-34	PNC-18-NO_2-17-N=NH_2-7-OCHO	93.06	16.57%
PNC-35	PNC-18-NO_2-17-N=NH_2-7-ONO	92.74	16.17%
PNC-36	PNC-18-NO_2-17-N=NH_2-7-OOH	92.66	16.07%
PNC-37	PNC-18-NO_2-17-N=NH_2-7-SH	91.83	15.03%
PNC-38	PNC-18-NO_2-17-N=NH_2-7-SO	92.78	16.22%
PNC-39	PNC-18-NO_2-17-N=NH_2-7-SO_2	92.86	16.32%
PNC-40	PNC-18-NO_2-17-N_3	88.80	11.24%
PNC-41	PNC-18-NO_2-17-N_3-7-N=NH_2	92.00	15.25%
PNC-42	PNC-18-NO_2-17-N_3-7-N_3	92.01	15.26%
PNC-43	PNC-18-NO_2-17-N_3-7-NH-NH_2	92.28	15.59%
PNC-44	PNC-18-NO_2-17-N_3-7-NO	92.75	16.19%
PNC-45	PNC-18-NO_2-17-N_3-7-NO_2	94.75	18.69%
PNC-46	PNC-18-NO_2-17-N_3-7-ONO	91.91	15.14%
PNC-47	PNC-18-NO_2-17-N_3-7-OOH	91.95	15.18%
PNC-48	PNC-18-NO_2-17-N_3-7-SH	91.01	14.00%

PNC-49	PNC-18-NO_2-17-N_3-7-SO	92.29	15.61%
PNC-50	PNC-18-NO_2-17-N_3-7-SO_2	92.86	16.32%
PNC-51	PNC-18-NO_2-17-NH-NH_2	90.20	13.00%
PNC-52	PNC-18-NO_2-17-NH-NH_2-7-N=NH_2	93.16	16.70%
PNC-53	PNC-18-NO_2-17-NH-NH_2-7-N_3	92.96	16.45%
PNC-54	PNC-18-NO_2-17-NH-NH_2-7-NO	93.29	16.86%
PNC-55	PNC-18-NO_2-17-NH-NH_2-7-NO_2	94.03	17.78%
PNC-56	PNC-18-NO_2-17-NH-NH_2-7-OCHO	93.02	16.52%
PNC-57	PNC-18-NO_2-17-NH-NH_2-7-ONO	92.80	16.25%
PNC-58	PNC-18-NO_2-17-NH-NH_2-7-OOH	92.91	16.39%
PNC-59	PNC-18-NO_2-17-NH-NH_2-7-SH	91.95	15.18%
PNC-60	PNC-18-NO_2-17-NH-NH_2-7-SO	93.02	16.52%
PNC-61	PNC-18-NO_2-17-NH-NH_2-7-SO_2	93.07	16.59%
PNC-62	PNC-18-NO_2-17-NO	94.33	18.16%
PNC-63	PNC-18-NO_2-17-NO-7-N=NH_2	94.05	17.82%
PNC-64	PNC-18-NO_2-17-NO-7-N_3	94.06	17.83%
PNC-65	PNC-18-NO_2-17-NO-7-NH-NH_2	94.16	17.95%

PNC-66	PNC-18-NO_2-17-NO-7-NO	94.86	18.82%
PNC-67	PNC-18-NO_2-17-NO-7-NO_2	96.82	21.28%
PNC-68	PNC-18-NO_2-17-NO-7-OCHO	94.24	18.05%
PNC-69	PNC-18-NO_2-17-NO-7-ONO	93.96	17.70%
PNC-70	PNC-18-NO_2-17-NO-7-OOH	93.97	17.72%
PNC-71	PNC-18-NO_2-17-NO-7-SH	93.08	16.60%
PNC-72	PNC-18-NO_2-17-NO-7-SO	94.43	18.29%
PNC-73	PNC-18-NO_2-17-NO-7-SO_2	95.00	19.00%
PNC-74	PNC-18-NO_2-17-NO_2	91.27	14.33%
PNC-75	PNC-18-NO_2-17-NO_2-7-N=NH_2	94.35	18.19%
PNC-76	PNC-18-NO_2-17-NO_2-7-NO	95.20	19.25%
PNC-77	PNC-18-NO_2-17-NO_2-7-NO_2	97.09	21.62%
PNC-78	PNC-18-NO_2-17-NO_2-7-OCHO	94.54	18.43%
PNC-79	PNC-18-NO_2-17-NO_2-7-ONO	94.24	18.05%
PNC-80	PNC-18-NO_2-17-NO_2-7-OOH	94.26	18.08%
PNC-81	PNC-18-NO_2-17-NO_2-7-SH	93.34	16.93%
PNC-82	PNC-18-NO_2-17-NO_2-7-SO_2	95.22	19.28%

PNC-83	PNC-18-NO_2-17-NO_2-7-N_3	94.32	18.15%
PNC-84	PNC-18-NO_2-17-NO_2-7-NH-NH_2	94.65	18.56%
PNC-85	PNC-18-NO_2-17-OCHO	89.43	12.03%
PNC-86	PNC-18-NO_2-17-ONO	88.69	11.10%
PNC-87	PNC-18-NO_2-17-OOH	89.17	11.70%
PNC-88	PNC-18-NO_2-17-SH	88.85	11.29%
PNC-89	PNC-18-NO_2-17-SO_2	94.60	18.50%
PNC-90	PNC-18-OCHO	92.50	15.87%
PNC-91	PNC-18-OCHO-17-NH-NH_2	85.73	7.39%
PNC-92	PNC-18-OCHO-17-NO_2	87.97	10.19%
PNC-93	PNC-18-OCHO-17-OCHO	85.84	7.52%
PNC-94	PNC-18-OCHO-17-OOH	85.51	7.12%
PNC-95	PNC-18-OCHO-17-SO	85.87	7.57%
PNC-96	PNC-18-OCHO-17-SO_2	86.32	8.13%
PNC-97	PNC-18-ONO	91.19	14.23%
PNC-98	PNC-18-ONO-17-NO_2	86.61	8.49%
PNC-99	PNC-18-OOH-17-NO_2	86.34	8.16%

PNC-100	PNC-18-SH	90.73	13.65%
PNC-101	PNC-18-SO	92.20	15.49%
PNC-102	PNC-18-SO-17-N=NH_2	92.21	15.51%
PNC-103	PNC-18-SO-17-NO	92.25	15.56%
PNC-104	PNC-18-SO-17-OCHO	92.50	15.88%
PNC-105	PNC-18-SO_2	93.35	16.94%
PNC-106	PNC-18-SO_2-17-N=NH_2	93.11	16.64%
PNC-107	PNC-18-SO_2-17-N=NH_2-7-N=NH_2	92.85	16.31%
PNC-108	PNC-18-SO_2-17-N=NH_2-7-N_3	92.60	16.00%
PNC-109	PNC-18-SO_2-17-N=NH_2-7-NH-NH_2	93.31	16.89%
PNC-110	PNC-18-SO_2-17-N=NH_2-7-NO	92.91	16.38%
PNC-111	PNC-18-SO_2-17-N=NH_2-7-NO_2	93.44	17.05%
PNC-112	PNC-18-SO_2-17-N=NH_2-7-OCHO	92.77	16.21%
PNC-113	PNC-18-SO_2-17-N=NH_2-7-ONO	92.49	15.86%
PNC-114	PNC-18-SO_2-17-N=NH_2-7-OOH	92.61	16.01%
PNC-115	PNC-18-SO_2-17-N=NH_2-7-SH	91.67	14.83%
PNC-116	PNC-18-SO_2-17-N=NH_2-7-SO	92.79	16.23%

PNC-117	PNC-18-SO_2-17-N=NH_2-7-SO_2	92.79	16.24%
PNC-118	PNC-18-SO_2-17-N_3	92.37	15.71%
PNC-119	PNC-18-SO_2-17-NH-NH_2	93.34	16.93%
PNC-120	PNC-18-SO_2-17-NH-NH_2-7-N=NH_2	93.12	16.64%
PNC-121	PNC-18-SO_2-17-NH-NH_2-7-N_3	92.88	16.35%
PNC-122	PNC-18-SO_2-17-NH-NH_2-7-NO	93.13	16.66%
PNC-123	PNC-18-SO_2-17-NH-NH_2-7-NO_2	93.64	17.30%
PNC-124	PNC-18-SO_2-17-NH-NH_2-7-OCHO	92.96	16.44%
PNC-125	PNC-18-SO_2-17-NH-NH_2-7-ONO	92.72	16.15%
PNC-126	PNC-18-SO_2-17-NH-NH_2-7-OOH	92.85	16.31%
PNC-127	PNC-18-SO_2-17-NH-NH_2-7-SH	91.80	14.99%
PNC-128	PNC-18-SO_2-17-NH-NH_2-7-SO	92.98	16.47%
PNC-129	PNC-18-SO_2-17-NH-NH_2-7-SO_2	92.99	16.48%
PNC-130	PNC-18-SO_2-17-NO	93.23	16.79%
PNC-131	PNC-18-SO_2-17-NO-7-N=NH_2	93.16	16.70%
PNC-132	PNC-18-SO_2-17-NO-7-N_3	92.96	16.45%
PNC-133	PNC-18-SO_2-17-NO-7-NH-NH_2	93.44	17.05%

PNC-134	PNC-18-SO_2-17-NO-7-NO	93.08	16.59%
PNC-135	PNC-18-SO_2-17-NO-7-NO_2	93.68	17.34%
PNC-136	PNC-18-SO_2-17-NO-7-OCHO	92.89	16.36%
PNC-137	PNC-18-SO_2-17-NO-7-ONO	92.62	16.02%
PNC-138	PNC-18-SO_2-17-NO-7-OOH	92.71	16.13%
PNC-139	PNC-18-SO_2-17-NO-7-SH	91.87	15.08%
PNC-140	PNC-18-SO_2-17-NO-7-SO	92.91	16.38%
PNC-141	PNC-18-SO_2-17-NO-7-SO_2	92.93	16.40%
PNC-142	PNC-18-SO_2-17-NO_2	91.04	14.04%
PNC-143	PNC-18-SO_2-17-OCHO	93.55	17.19%
PNC-144	PNC-18-SO_2-17-OCHO-7-N=NH_2	93.42	17.02%
PNC-145	PNC-18-SO_2-17-OCHO-7-N_3	93.21	16.76%
PNC-146	PNC-18-SO_2-17-OCHO-7-NH-NH_2	93.88	17.60%
PNC-147	PNC-18-SO_2-17-OCHO-7-NO	93.47	17.09%
PNC-148	PNC-18-SO_2-17-OCHO-7-NO_2	95.02	19.03%
PNC-149	PNC-18-SO_2-17-OCHO-7-OCHO	93.66	17.32%
PNC-150	PNC-18-SO_2-17-OCHO-7-ONO	93.14	16.67%

PNC-151	PNC-18-SO_2-17-OCHO-7-OOH	93.07	16.58%
PNC-152	PNC-18-SO_2-17-OCHO-7-SH	92.12	15.40%
PNC-153	PNC-18-SO_2-17-OCHO-7-SO	93.28	16.84%
PNC-154	PNC-18-SO_2-17-OCHO-7-SO_2	93.25	16.81%
PNC-155	PNC-18-SO_2-17-ONO	90.46	13.31%
PNC-156	PNC-18-SO_2-17-OOH	93.52	17.15%
PNC-157	PNC-18-SO_2-17-OOH-7-N=NH_2	93.41	17.01%
PNC-158	PNC-18-SO_2-17-OOH-7-N_3	93.17	16.70%
PNC-159	PNC-18-SO_2-17-OOH-7-NH-NH_2	93.91	17.64%
PNC-160	PNC-18-SO_2-17-OOH-7-NO	93.26	16.82%
PNC-161	PNC-18-SO_2-17-OOH-7-NO_2	93.58	17.22%
PNC-162	PNC-18-SO_2-17-OOH-7-OCHO	93.24	16.80%
PNC-163	PNC-18-SO_2-17-OOH-7-ONO	92.90	16.38%
PNC-164	PNC-18-SO_2-17-OOH-7-OOH	93.04	16.54%
PNC-165	PNC-18-SO_2-17-OOH-7-SH	92.10	15.36%
PNC-166	PNC-18-SO_2-17-SH	92.83	16.29%
PNC-167	PNC-18-SO_2-17-SH-7-N=NH_2	92.72	16.15%

PNC-168	PNC-18-SO_2-17-SH-7-N_3	92.52	15.90%
PNC-169	PNC-18-SO_2-17-SH-7-NH-NH_2	93.21	16.76%
PNC-170	PNC-18-SO_2-17-SH-7-NO	93.28	16.84%
PNC-171	PNC-18-SO_2-17-SH-7-NO_2	94.79	18.74%
PNC-172	PNC-18-SO_2-17-SH-7-OCHO	92.94	16.42%
PNC-173	PNC-18-SO_2-17-SH-7-ONO	92.43	15.79%
PNC-174	PNC-18-SO_2-17-SH-7-OOH	92.42	15.77%
PNC-175	PNC-18-SO_2-17-SH-7-SH	91.38	14.47%
PNC-176	PNC-18-SO_2-17-SH-7-SO_2	93.37	16.96%
PNC-177	PNC-18-SO_2-17-SO-7-N=NH_2	93.31	16.89%
PNC-178	PNC-18-SO_2-17-SO-7-N_3	93.12	16.65%
PNC-179	PNC-18-SO_2-17-SO-7-NH-NH_2	93.85	17.56%
PNC-180	PNC-18-SO_2-17-SO-7-NO	93.44	17.05%
PNC-181	PNC-18-SO_2-17-SO-7-OCHO	93.23	16.78%
PNC-182	PNC-18-SO_2-17-SO-7-ONO	93.00	16.49%
PNC-183	PNC-18-SO_2-17-SO_2	93.78	17.48%
PNC-184	PNC-18-SO_2-17-SO_2-7-N=NH_2	93.63	17.29%

PNC-185	PNC-18-SO_2-17-SO_2-7-N_3	93.43	17.04%
PNC-186	PNC-18-SO_2-17-SO_2-7-NH-NH_2	94.11	17.88%
PNC-187	PNC-18-SO_2-17-SO_2-7-NO	93.74	17.43%
PNC-188	PNC-18-SO_2-17-SO_2-7-NO_2	94.42	18.28%
PNC-189	PNC-18-SO_2-17-SO_2-7-OCHO	93.74	17.42%
PNC-190	PNC-18-SO_2-17-SO_2-7-ONO	93.30	16.88%
PNC-191	PNC-18-SO_2-17-SO_2-7-OOH	93.40	17.00%
PNC-192	PNC-18-SO_2-17-SO_2-7-SH	92.42	15.77%
PNC-193	PNC-18-SO_2-17-SO_2-7-SO_2	93.59	17.24%
PNC-194	PNC-7-NH-NH_2	91.20	14.24%
PNC-195	PNC-7-NO	91.73	14.91%
PNC-196	PNC-7-OOH	90.60	13.49%
PNC-197	PNC-7-OCHO	91.24	14.29%
PNC-198	PNC-7-ONO	90.77	13.71%
PNC-199	PNC-7-SH	89.56	12.19%
PNC-200	PNC-7-SO	90.70	13.62%
PNC-201	PNC-7-SO_2	90.77	13.70%

Table S3 Evaluation of environmental friendliness of β-lactam substitutes.

No.	Compounds	LogK_{ow}	Change rate (%)	LC_{50} (mg/L)	Change rate (%)	LogK_{oa}
-	PNC	1.83	-	1282.44	-	14.15
PNC-1	PNC-18-N=NH_2	3.20	74.86	152.83	-88.08	16.00
PNC-2	PNC-18-N=NH_2-17-N_3	4.08	122.95	40.02	-96.88	15.78
PNC-3	PNC-18-N=NH_2-17-NH-NH_2	1.36	-25.68	3387.43	164.14	15.32
PNC-4	PNC-18-N=NH_2-17-NO	3.01	64.48	223.97	-82.54	15.83
PNC-5	PNC-18-N=NH_2-17-NO_2	2.33	27.32	717.72	-44.03	15.64
PNC-6	PNC-18-N=NH_2-17-OCHO	2.19	19.67	897.64	-30.01	15.55
PNC-7	PNC-18-N=NH_2-17-ONO	3.25	77.60	158.18	-87.67	15.58
PNC-8	PNC-18-N=NH_2-17-OOH	1.94	6.01	1314.27	2.48	16.02
PNC-9	PNC-18-N=NH_2-17-SH	3.14	71.58	182.26	-85.79	15.82
PNC-10	PNC-18-N=NH_2-17-SO	0.47	-74.32	15447.41	1104.53	15.62
PNC-11	PNC-18-N=NH_2-17-SO_2	0.58	-68.31	13238.89	932.32	15.37
PNC-12	PNC-18-N=NH_2-7-N=NH_2	3.42	86.89	113.65	-91.14	15.73
PNC-13	PNC-18-N=NH_2-7-N_3	4.00	118.58	45.17	-96.48	15.82

PNC-14	PNC-18-N=NH_2-7-NH-NH_2	1.29	-29.51	3823.23	198.12	16.23
PNC-15	PNC-18-N=NH_2-7-NO	2.94	60.66	252.78	-80.29	15.93
PNC-16	PNC-18-N=NH_2-7-NO_2	3.26	78.14	156.11	-87.83	15.91
PNC-17	PNC-18-N=NH_2-7-OCHO	2.62	43.17	444.74	-65.32	16.09
PNC-18	PNC-18-N=NH_2-7-OOH	1.87	2.19	1483.35	15.67	15.96
PNC-19	PNC-18-N=NH_2-7-SH	3.07	67.76	205.71	-83.96	15.88
PNC-20	PNC-18-N=NH_2-7-SO	1.13	-38.25	5150.06	301.58	15.98
PNC-21	PNC-18-N=NH_2-7-SO_2	1.25	-31.69	4413.76	244.17	15.98
PNC-22	PNC-18-N_3	3.78	106.56	60.88	-95.25	15.75
PNC-23	PNC-18-NH-NH_2	1.07	-41.53	5143.29	301.05	15.86
PNC-24	PNC-18-NH-NH_2-17-NO_2	-0.86	-146.99	135000.00	10426.80	17.23
PNC-25	PNC-18-NH-NH_2-17-SO_2	-2.60	-242.08	2490000.00	194060.98	17.75
PNC-26	PNC-18-NO	2.72	48.63	340.00	-73.49	15.82
PNC-27	PNC-18-NO_2	2.03	10.93	1092.73	-14.79	15.48
PNC-28	PNC-18-NO_2-17-N=NH_2	1.28	-30.05	4003.92	212.21	18.25
PNC-29	PNC-18-NO_2-17-N=NH_2-7-N=NH_2	2.55	39.34	529.61	-58.70	15.94
PNC-30	PNC-18-NO_2-17-N=NH_2-7-N_3	3.13	71.04	209.80	-83.64	16.18

PNC-31	PNC-18-NO_2-17-N=NH_2-7-NH-NH_2	0.42	-77.05	17807.49	1288.56	16.27
PNC-32	PNC-18-NO_2-17-N=NH_2-7-NO	2.07	13.11	1177.70	-8.17	15.99
PNC-33	PNC-18-NO_2-17-N=NH_2-7-NO_2	2.39	30.60	724.42	-43.51	15.99
PNC-34	PNC-18-NO_2-17-N=NH_2-7-OCHO	1.75	-4.37	2064.37	60.97	16.09
PNC-35	PNC-18-NO_2-17-N=NH_2-7-ONO	2.31	26.23	828.46	-35.40	16.03
PNC-36	PNC-18-NO_2-17-N=NH_2-7-OOH	1.00	-45.36	6905.57	438.47	15.79
PNC-37	PNC-18-NO_2-17-N=NH_2-7-SH	2.20	20.22	957.66	-25.33	15.83
PNC-38	PNC-18-NO_2-17-N=NH_2-7-SO	0.26	-85.79	23893.47	1763.12	15.85
PNC-39	PNC-18-NO_2-17-N=NH_2-7-SO_2	0.38	-79.23	20402.89	1490.94	16.01
PNC-40	PNC-18-NO_2-17-N_3	1.86	1.64	1589.89	23.97	18.01
PNC-41	PNC-18-NO_2-17-N_3-7-N=NH_2	3.13	71.04	209.80	-83.64	15.56
PNC-42	PNC-18-NO_2-17-N_3-7-N_3	3.71	102.73	83.04	-93.52	15.68
PNC-43	PNC-18-NO_2-17-N_3-7-NH-NH_2	1.00	-45.36	7053.28	449.99	15.82
PNC-44	PNC-18-NO_2-17-N_3-7-NO	2.65	44.81	466.50	-63.62	15.49
PNC-45	PNC-18-NO_2-17-N_3-7-NO_2	2.97	62.30	286.67	-77.65	15.42
PNC-46	PNC-18-NO_2-17-N_3-7-ONO	2.89	57.92	327.84	-74.44	15.50
PNC-47	PNC-18-NO_2-17-N_3-7-OOH	1.58	-13.66	2734.85	113.25	15.11

PNC-48	PNC-18-NO_2-17-N_3-7-SH	2.78	51.91	379.27	-70.43	15.34
PNC-49	PNC-18-NO_2-17-N_3-7-SO	0.84	-54.10	9454.45	637.22	15.71
PNC-50	PNC-18-NO_2-17-N_3-7-SO_2	0.96	-47.54	8065.79	528.94	15.57
PNC-51	PNC-18-NO_2-17-NH-NH_2	-0.86	-146.99	13500000.00	1052580.01	18.04
PNC-52	PNC-18-NO_2-17-NH-NH_2-7-N=NH_2	0.42	-77.05	17807.49	1288.56	15.19
PNC-53	PNC-18-NO_2-17-NH-NH_2-7-N_3	1.00	-45.36	7053.28	449.99	15.27
PNC-54	PNC-18-NO_2-17-NH-NH_2-7-NO	-0.06	-103.28	39598.10	2987.71	15.39
PNC-55	PNC-18-NO_2-17-NH-NH_2-7-NO_2	0.25	-86.34	24353.46	1798.99	15.43
PNC-56	PNC-18-NO_2-17-NH-NH_2-7-OCHO	-0.38	-120.77	69400.31	5311.58	15.84
PNC-57	PNC-18-NO_2-17-NH-NH_2-7-ONO	0.17	-90.71	27851.03	2071.72	15.59
PNC-58	PNC-18-NO_2-17-NH-NH_2-7-OOH	-1.13	-161.75	232000.00	17990.50	15.55
PNC-59	PNC-18-NO_2-17-NH-NH_2-7-SH	0.07	-96.17	32198.52	2410.72	15.45
PNC-60	PNC-18-NO_2-17-NH-NH_2-7-SO	-1.87	-202.19	803000.00	62514.97	15.56
PNC-61	PNC-18-NO_2-17-NH-NH_2-7-SO_2	-1.75	-195.63	686000.00	53391.74	15.64
PNC-62	PNC-18-NO_2-17-NO	1.85	1.09	1596.24	24.47	15.92
PNC-63	PNC-18-NO_2-17-NO-7-N=NH_2	2.07	13.11	1177.70	-8.17	15.89
PNC-64	PNC-18-NO_2-17-NO-7-N_3	2.65	44.81	466.50	-63.62	16.07

PNC-65	PNC-18-NO_2-17-NO-7-NH-NH_2	-0.06	-103.28	39598.10	2987.71	16.10
PNC-66	PNC-18-NO_2-17-NO-7-NO	1.59	-13.11	2618.84	104.21	15.90
PNC-67	PNC-18-NO_2-17-NO-7-NO_2	1.90	3.83	1610.76	25.60	15.80
PNC-68	PNC-18-NO_2-17-NO-7-OCHO	1.27	-30.60	4590.18	257.92	16.07
PNC-69	PNC-18-NO_2-17-NO-7-ONO	1.82	-0.55	1842.09	43.64	16.01
PNC-70	PNC-18-NO_2-17-NO-7-OOH	0.52	-71.58	15355.60	1097.37	15.81
PNC-71	PNC-18-NO_2-17-NO-7-SH	1.72	-6.01	2129.50	66.05	15.83
PNC-72	PNC-18-NO_2-17-NO-7-SO	-0.22	-112.02	53127.25	4042.67	16.12
PNC-73	PNC-18-NO_2-17-NO-7-SO_2	-0.10	-105.46	45362.66	3437.21	16.02
PNC-74	PNC-18-NO_2-17-NO_2	0.11	-93.99	28507.51	2122.91	18.46
PNC-75	PNC-18-NO_2-17-NO_2-7-N=NH_2	1.39	-24.04	3759.16	193.13	15.90
PNC-76	PNC-18-NO_2-17-NO_2-7-NO	0.90	-50.82	8358.54	551.77	15.86
PNC-77	PNC-18-NO_2-17-NO_2-7-NO_2	1.22	-33.33	5134.82	300.39	15.72
PNC-78	PNC-18-NO_2-17-NO_2-7-OCHO	0.58	-68.31	14633.73	1041.08	16.10
PNC-79	PNC-18-NO_2-17-NO_2-7-ONO	1.14	-37.70	3364.52	162.35	15.97
PNC-80	PNC-18-NO_2-17-NO_2-7-OOH	-0.17	-109.29	48998.90	3720.75	15.70
PNC-81	PNC-18-NO_2-17-NO_2-7-SH	1.03	-43.72	6795.09	429.86	15.81

PNC-82	PNC-18-NO_2-17-NO_2-7-SO_2	-0.79	-143.17	144000.00	11128.59	15.97
PNC-83	PNC-18-NO_2-17-NO_2-7-N_3	1.97	7.65	1487.56	15.99	16.03
PNC-84	PNC-18-NO_2-17-NO_2-7-NH-NH_2	-0.75	-140.98	126000.00	9725.01	16.22
PNC-85	PNC-18-NO_2-17-OCHO	-0.03	-101.64	35655.84	2680.31	18.82
PNC-86	PNC-18-NO_2-17-ONO	1.03	-43.72	6282.61	389.89	18.48
PNC-87	PNC-18-NO_2-17-OOH	-0.28	-115.30	52245.61	3973.92	18.85
PNC-88	PNC-18-NO_2-17-SH	0.92	-49.73	7245.46	464.97	18.43
PNC-89	PNC-18-NO_2-17-SO_2	-0.58	-131.69	94057.26	7234.24	15.27
PNC-90	PNC-18-OCHO	1.89	3.28	1366.42	6.55	16.15
PNC-91	PNC-18-OCHO-17-NH-NH_2	-0.99	-154.10	168000.00	13000.02	19.34
PNC-92	PNC-18-OCHO-17-NO_2	-0.03	-101.64	356555.84	27702.90	18.84
PNC-93	PNC-18-OCHO-17-OCHO	-0.17	-109.29	44596.39	3377.46	19.46
PNC-94	PNC-18-OCHO-17-OOH	-0.41	-122.40	65341.52	4995.09	19.14
PNC-95	PNC-18-OCHO-17-SO	-1.89	-203.28	767000.00	59707.82	19.48
PNC-96	PNC-18-OCHO-17-SO_2	-1.77	-196.72	657000.00	51130.43	19.58
PNC-97	PNC-18-ONO	2.95	61.20	240.82	-81.22	16.02
PNC-98	PNC-18-ONO-17-NO_2	1.03	-43.72	6282.61	389.89	19.01

PNC-99	PNC-18-OOH-17-NO_2	-0.28	-115.30	52245.61	3973.92	18.56
PNC-100	PNC-18-SH	2.84	55.19	276.85	-78.41	15.76
PNC-101	PNC-18-SO	0.17	-90.71	23523.05	1734.24	16.03
PNC-102	PNC-18-SO-17-N=NH_2	-1.66	-190.71	520000.00	40447.67	15.92
PNC-103	PNC-18-SO-17-NO	-0.02	-101.09	34355.36	2578.90	16.38
PNC-104	PNC-18-SO-17-OCHO	-0.84	-145.90	137000.00	10582.75	15.93
PNC-105	PNC-18-SO_2	0.29	-84.15	20214.26	1476.23	16.53
PNC-106	PNC-18-SO_2-17-N=NH_2	0.58	-68.31	13238.89	932.32	16.09
PNC-107	PNC-18-SO_2-17-N=NH_2-7-N=NH_2	0.81	-55.74	9744.56	659.84	16.30
PNC-108	PNC-18-SO_2-17-N=NH_2-7-N_3	1.39	-24.04	3856.07	200.68	16.24
PNC-109	PNC-18-SO_2-17-N=NH_2-7-NH-NH_2	-1.32	-172.13	328000.00	25476.23	16.54
PNC-110	PNC-18-SO_2-17-N=NH_2-7-NO	0.32	-82.51	21667.17	1589.53	15.75
PNC-111	PNC-18-SO_2-17-N=NH_2-7-NO_2	0.64	-65.03	13310.51	937.90	15.83
PNC-112	PNC-18-SO_2-17-N=NH_2-7-OCHO	0.00	-100.00	37933.68	2857.93	16.19
PNC-113	PNC-18-SO_2-17-N=NH_2-7-ONO	0.56	-69.40	15222.13	1086.97	15.89
PNC-114	PNC-18-SO_2-17-N=NH_2-7-OOH	-0.75	-140.98	127000.00	9802.99	15.92
PNC-115	PNC-18-SO_2-17-N=NH_2-7-SH	0.45	-75.41	17614.35	1273.50	15.92

PNC-116	PNC-18-SO_2-17-N=NH_2-7-SO	-1.48	-180.87	439000.00	34131.59	16.05
PNC-117	PNC-18-SO_2-17-N=NH_2-7-SO_2	0.47	-74.32	18419.45	1336.28	16.01
PNC-118	PNC-18-SO_2-17-N_3	1.16	-36.61	5248.23	309.24	16.59
PNC-119	PNC-18-SO_2-17-NH-NH_2	-1.55	-184.70	445000.00	34599.45	16.45
PNC-120	PNC-18-SO_2-17-NH-NH_2-7-N=NH_2	-1.32	-172.13	328000.00	25476.23	16.10
PNC-121	PNC-18-SO_2-17-NH-NH_2-7-N_3	-0.74	-140.44	130000.00	10036.92	16.03
PNC-122	PNC-18-SO_2-17-NH-NH_2-7-NO	-1.81	-198.91	728000.00	56666.74	16.18
PNC-123	PNC-18-SO_2-17-NH-NH_2-7-NO_2	-1.49	-181.42	447000.00	34755.40	16.20
PNC-124	PNC-18-SO_2-17-NH-NH_2-7-OCHO	-2.13	-216.39	1280000.00	99709.66	16.68
PNC-125	PNC-18-SO_2-17-NH-NH_2-7-ONO	-1.57	-185.79	512000.00	39823.86	16.23
PNC-126	PNC-18-SO_2-17-NH-NH_2-7-OOH	-2.88	-257.38	4270000.00	332858.79	16.28
PNC-127	PNC-18-SO_2-17-NH-NH_2-7-SH	-1.68	-191.80	592000.00	46061.97	16.16
PNC-128	PNC-18-SO_2-17-NH-NH_2-7-SO	-3.61	-297.27	14800000.00	1153949.19	16.44
PNC-129	PNC-18-SO_2-17-NH-NH_2-7-SO_2	-1.67	-191.26	619000.00	48167.33	16.45
PNC-130	PNC-18-SO_2-17-NO	0.10	-94.54	29440.98	2195.70	16.04
PNC-131	PNC-18-SO_2-17-NO-7-N=NH_2	0.32	-82.51	21667.17	1589.53	16.22
PNC-132	PNC-18-SO_2-17-NO-7-N_3	0.90	-50.82	8573.53	568.53	16.36

PNC-133	PNC-18-SO_2-17-NO-7-NH-NH_2	-1.81	-198.91	728000.00	56666.74	16.39
PNC-134	PNC-18-SO_2-17-NO-7-NO	-0.16	-108.74	48177.07	3656.67	15.91
PNC-135	PNC-18-SO_2-17-NO-7-NO_2	0.16	-91.26	29593.86	2207.62	15.93
PNC-136	PNC-18-SO_2-17-NO-7-OCHO	-0.48	-126.23	84340.01	6476.52	16.21
PNC-137	PNC-18-SO_2-17-NO-7-ONO	0.08	-95.63	33844.04	2539.03	16.09
PNC-138	PNC-18-SO_2-17-NO-7-OOH	-1.23	-167.21	282000.00	21889.32	15.97
PNC-139	PNC-18-SO_2-17-NO-7-SH	-0.03	-101.64	39165.04	2953.95	16.01
PNC-140	PNC-18-SO_2-17-NO-7-SO	-1.96	-207.10	976000.00	76004.87	16.02
PNC-141	PNC-18-SO_2-17-NO-7-SO_2	-0.02	-101.09	40949.86	3093.12	16.06
PNC-142	PNC-18-SO_2-17-NO_2	-0.58	-131.69	94057.26	7234.24	16.94
PNC-143	PNC-18-SO_2-17-OCHO	-0.72	-139.34	118000.00	9101.20	15.76
PNC-144	PNC-18-SO_2-17-OCHO-7-N=NH_2	-0.50	-127.32	86412.18	6638.10	16.10
PNC-145	PNC-18-SO_2-17-OCHO-7-N_3	0.08	-95.63	34162.85	2563.89	16.16
PNC-146	PNC-18-SO_2-17-OCHO-7-NH-NH_2	-2.63	-243.72	2900000.00	226031.26	16.11
PNC-147	PNC-18-SO_2-17-OCHO-7-NO	-0.98	-153.55	192000.00	14871.45	15.75
PNC-148	PNC-18-SO_2-17-OCHO-7-NO_2	-0.66	-136.07	118000.00	9101.20	15.74
PNC-149	PNC-18-SO_2-17-OCHO-7-OCHO	-1.30	-171.04	336000.00	26100.04	16.13

PNC-150	PNC-18-SO_2-17-OCHO-7-ONO	-0.74	-140.44	135000.00	10426.80	16.07
PNC-151	PNC-18-SO_2-17-OCHO-7-OOH	-2.05	-212.02	1130000.00	88013.22	15.61
PNC-152	PNC-18-SO_2-17-OCHO-7-SH	-0.85	-146.45	156000.00	12064.30	15.60
PNC-153	PNC-18-SO_2-17-OCHO-7-SO	-2.79	-252.46	3890000.00	303227.79	15.81
PNC-154	PNC-18-SO_2-17-OCHO-7-SO_2	-0.84	-145.90	163000.00	12610.14	15.79
PNC-155	PNC-18-SO_2-17-ONO	0.34	-81.42	20728.74	1516.35	16.85
PNC-156	PNC-18-SO_2-17-OOH	-0.97	-153.01	173000.00	13389.90	15.74
PNC-157	PNC-18-SO_2-17-OOH-7-N=NH_2	-0.75	-140.98	127000.00	9802.99	16.11
PNC-158	PNC-18-SO_2-17-OOH-7-N_3	-0.17	-109.29	50250.15	3818.32	16.14
PNC-159	PNC-18-SO_2-17-OOH-7-NH-NH_2	-2.88	-257.38	4270000.00	332858.79	16.10
PNC-160	PNC-18-SO_2-17-OOH-7-NO	-1.23	-167.21	282000.00	21889.32	15.45
PNC-161	PNC-18-SO_2-17-OOH-7-NO_2	-0.91	-149.73	173000.00	13389.90	15.47
PNC-162	PNC-18-SO_2-17-OOH-7-OCHO	-1.55	-184.70	494000.00	38420.29	16.06
PNC-163	PNC-18-SO_2-17-OOH-7-ONO	-0.99	-154.10	198000.00	15339.31	15.55
PNC-164	PNC-18-SO_2-17-OOH-7-OOH	-2.30	-225.68	1660000.00	129340.65	15.60
PNC-165	PNC-18-SO_2-17-OOH-7-SH	-1.10	-160.11	230000.00	17834.55	15.57
PNC-166	PNC-18-SO_2-17-SH	0.23	-87.43	23944.36	1767.09	15.99

PNC-167	PNC-18-SO_2-17-SH-7-N=NH_2	0.45	-75.41	17614.35	1273.50	16.22
PNC-168	PNC-18-SO_2-17-SH-7-N_3	1.03	-43.72	6968.59	443.38	16.33
PNC-169	PNC-18-SO_2-17-SH-7-NH-NH_2	-1.68	-191.80	592000.00	46061.97	16.29
PNC-170	PNC-18-SO_2-17-SH-7-NO	-0.03	-101.64	39165.04	2953.95	16.09
PNC-171	PNC-18-SO_2-17-SH-7-NO_2	0.29	-84.15	24052.68	1775.54	16.04
PNC-172	PNC-18-SO_2-17-SH-7-OCHO	-0.35	-119.13	68549.04	5245.20	16.28
PNC-173	PNC-18-SO_2-17-SH-7-ONO	0.21	-88.52	27507.06	2044.90	16.24
PNC-174	PNC-18-SO_2-17-SH-7-OOH	-1.10	-160.11	230000.00	17834.55	16.15
PNC-175	PNC-18-SO_2-17-SH-7-SH	0.10	-94.54	31837.42	2382.56	16.03
PNC-176	PNC-18-SO_2-17-SH-7-SO_2	0.11	-93.99	33275.04	2494.66	16.24
PNC-177	PNC-18-SO_2-17-SO-7-N=NH_2	-2.22	-221.31	1490000.00	116084.68	15.87
PNC-178	PNC-18-SO_2-17-SO-7-N_3	-1.64	-189.62	587000.00	45672.09	15.89
PNC-179	PNC-18-SO_2-17-SO-7-NH-NH_2	-4.35	-337.70	50000000.00	3898714.84	16.67
PNC-180	PNC-18-SO_2-17-SO-7-NO	-2.71	-248.09	3300000.00	257221.78	15.93
PNC-181	PNC-18-SO_2-17-SO-7-OCHO	-3.03	-265.57	5780000.00	450603.00	16.33
PNC-182	PNC-18-SO_2-17-SO-7-ONO	-2.47	-234.97	2320000.00	180805.01	16.08
PNC-183	PNC-18-SO_2-17-SO_2	-0.50	-127.32	85097.58	6535.59	15.85

PNC-184	PNC-18-SO_2-17-SO_2-7-N=NH_2	-0.28	-115.30	62356.21	4762.31	15.54
PNC-185	PNC-18-SO_2-17-SO_2-7-N_3	0.30	-83.61	24628.31	1820.42	15.56
PNC-186	PNC-18-SO_2-17-SO_2-7-NH-NH_2	-2.41	-231.69	2100000.00	163650.22	16.16
PNC-187	PNC-18-SO_2-17-SO_2-7-NO	-0.76	-141.53	139000.00	10738.71	15.59
PNC-188	PNC-18-SO_2-17-SO_2-7-NO_2	-0.44	-124.04	84964.83	6525.24	15.64
PNC-189	PNC-18-SO_2-17-SO_2-7-OCHO	-1.08	-159.02	242000.00	18770.26	16.02
PNC-190	PNC-18-SO_2-17-SO_2-7-ONO	-0.52	-128.42	97167.23	7476.74	15.75
PNC-191	PNC-18-SO_2-17-SO_2-7-OOH	-1.83	-200.00	812000.00	63216.75	15.70
PNC-192	PNC-18-SO_2-17-SO_2-7-SH	-0.63	-134.43	113000.00	8711.32	15.65
PNC-193	PNC-18-SO_2-17-SO_2-7-SO_2	-0.62	-133.88	117000.00	9023.23	15.85
PNC-194	PNC-7-NH-NH_2	0.99	-45.90	5804.99	352.65	15.86
PNC-195	PNC-7-NO	2.64	44.26	383.74	-70.08	16.13
PNC-196	PNC-7-OOH	1.57	-14.21	2253.08	75.69	15.55
PNC-197	PNC-7-OCHO	2.32	26.78	677.01	-47.21	15.85
PNC-198	PNC-7-ONO	2.88	57.38	271.80	-78.81	16.14
PNC-199	PNC-7-SH	2.77	51.37	312.46	-75.64	15.46
PNC-200	PNC-7-SO	0.84	-54.10	7842.43	511.52	15.58

PNC-201	PNC-7-SO_2	0.95	-48.09	6739.30	425.51	15.58
